# Characteristics of Eyes Developing Retinal Detachment After Anti-vascular Endothelial Growth Factor Therapy for Retinopathy of Prematurity

**DOI:** 10.3389/fped.2022.785292

**Published:** 2022-04-07

**Authors:** Chiori Kondo, Chiharu Iwahashi, Shoko Utamura, Kazuki Kuniyoshi, Yuhei Konishi, Norihisa Wada, Ryo Kawasaki, Shunji Kusaka

**Affiliations:** ^1^Department of Ophthalmology, Kindai University Faculty of Medicine, Osakasayama, Japan; ^2^Department of Pediatrics, Kindai University Faculty of Medicine, Osakasayama, Japan; ^3^Department of Vision Informatics, Graduate School of Medicine, Osaka University, Suita, Osaka, Japan

**Keywords:** vascular endothelial growth factor, anti-vascular endothelial growth factor, retinopathy of prematurity, reactivation, retinal detachment, vitrectomy

## Abstract

**Background:**

We investigated the incidence and clinical characteristics of eyes showing retinal detachment (RD) after anti-vascular endothelial growth factor (VEGF) for retinopathy of prematurity (ROP).

**Methods:**

A retrospective chart review of 76 consecutive eyes of 45 patients (18 girls and 27 boys) with stage 3 ROP who received anti-VEGF therapy between January 2012 and August 2020 with a minimum follow-up of 6 months was conducted. Eyes were divided into two groups: the vitrectomy (V) group that required vitrectomy for RD after anti-VEGF therapy and the non-vitrectomy (non-V) group that did not require vitrectomy. Data were collected from patient charts, including sex, postmenstrual age (PMA) at birth, birth weight, PMA at anti-VEGF therapy, comorbidities, reactivation, examination interval, and subsequent vitrectomies.

**Results:**

The median PMA at birth was 24.7 (range, 22.1–29.3) weeks. Twenty-seven eyes (35.1%) exhibited ROP reactivation at 6.4 ± 3.1 weeks after anti-VEGF therapy. The V group included six eyes of five patients, all of whom exhibited reactivation and developed RD 10.1 ± 6.5 weeks after anti-VEGF therapy. The types of RD were conventional (classic) in two eyes and circumferential (unique to RD after anti-VEGF) in four eyes. Three eyes required repeated vitrectomy. All eyes, except one eye in the V group, achieved retinal attachment at the last examination. The non-V group included 70 eyes of 40 patients, of which 21 exhibited reactivation and were treated successfully with laser (17 eyes) or second anti-VEGF (4 eyes). The proportion of eyes with plus disease was significantly higher in the V group (50.0%) than in the non-V group (10.0%) (*P* = 0.035). V group included 3 of 22 eyes (13.6%) in which the interval between the last examination and the diagnosis of reactivation was <1 week and 3 of 5 eyes (60.0%) in which the interval was more than 1 week (*P* = 0.024). The two groups showed no significant differences in the other factors.

**Conclusion:**

Approximately 8% of eyes developed RD about 10 weeks after anti-VEGF therapy for ROP. Eyes with history of plus disease should be carefully monitored at appropriate intervals after anti-VEGF therapy for ROP.

## Introduction

Retinopathy of prematurity (ROP), which is caused by abnormal development of the retinal vessels of preterm infants (1), is the leading cause of infant blindness in both developed and developing countries (2). Over the past few decades, the standard treatment for avascular immature retinas has been laser ablation in patients with treatment-requiring ROP (3). However, the use of intravitreal injections of anti-vascular endothelial growth factor (anti-VEGF) agents has recently gained prominence. Bevacizumab (Avastin; Genentech Inc., South San Francisco, CA) and ranibizumab (Lucentis; Genentech Inc.) are the commonly used anti-VEGF drugs for these cases, and intravitreal injections of bevacizumab (IVB) and ranibizumab (IVR) have demonstrated efficacy in the treatment of stage 3 ROP (4–7). However, ROP reactivation after anti-VEGF therapy is not uncommon. Previous studies assessing the clinical outcomes of IVB or IVR therapy in eyes with ROP have reported reactivation rates of 6–14% (4, 7–11) and 0–80% (5, 7, 8, 12–14), respectively. Thus, timely detection and management of reactivation are major considerations for anti-VEGF therapy in cases of ROP.

Eyes with reactivation often present with recurrent plus disease or recurrent stage 3 ROP and may require treatment with laser ablation or repeated anti-VEGF therapy. Some eyes may progress to stage 4 or higher ROP, which may include, in addition to the typical traction RD seen in ROP, posterior atypical RD caused by fibrovascular contraction (15–19). The previous studies describing reactivation after anti-VEGF therapy focused on the reactivation rate and the risk factors for reactivation (7, 9, 12, 13, 20). However, to date, information regarding the characteristics and treatment outcomes of more severe cases showing RD development and requiring vitrectomy after IVB or IVR has not been well-documented.

Thus, the purpose of this study was to report the incidence, clinical characteristics, and treatment outcomes of eyes with RD after anti-VEGF therapy for ROP. We also examined the factors associated with the eyes that required vitrectomy.

## Methods

The study was approved by the Institutional Review Board of Kindai University Hospital (#26-251) and adhered to the tenets of the Declaration of Helsinki.

### Patients

The medical records of consecutive patients with stage 3 ROP who were treated with IVB or IVR at Kindai University Hospital, a tertiary referral pediatric retina center, between January 2012 and August 2020 were retrospectively reviewed. One patient who received IVB at a referring hospital and subsequently underwent vitrectomy at Kindai University Hospital was also included. Patients were excluded if they received anti-VEGF therapy as adjunctive therapy before planned vitrectomy or underwent follow-up assessments for <6 months. Patients were also excluded if they received anti-VEGF therapy between April 2018 and November 2019, because the procedures performed during that period were not approved by the institutional review board due to policy changes in the Clinical Trial Act in Japan.

### Ocular Examinations

At the initial examination, fundus photographs, and fluorescein angiograms were taken with a RetCam 3 digital fundus camera (Natus, San Carlo, CA, USA). The ROP stage and zone were evaluated by two pediatric retinal specialists based on the International Classification of Retinopathy of Prematurity, Third Edition (21). Ophthalmic examinations were performed before and 1, 7, 14, and 28 days after IVB or IVR therapy at our hospital and biweekly or monthly thereafter at the referring hospitals, depending on the fundus findings and systemic conditions. The efficacy of anti-VEGF therapy was evaluated by assessing improvements in the tortuosity and dilation of the retinal vessels and the dilation of the tunica vasculosa lentis. Reactivation was defined by the reappearance of vascular dilation, tortuosity, or new/recurrent neovascularization that required further treatment.

### Intravitreal Injections of Anti-vascular Endothelial Growth Factor

The choice of IVB or IVR was dependent on the treatment period. Patients treated between January 2012 and June 2015 received IVB (0.25 mg/0.01 mL), and those treated between July 2015 and March 2018 received IVR (0.25 mg/0.025 mL). Since ranibizumab 0.2 mg/0.02 mL was approved by the Japanese Pharmaceuticals and Medical Devices Agency for the treatment of ROP in November 2019, the dosage of ranibizumab was changed thereafter. The anti-VEGF agent was administered as monotherapy for treatment-naïve patients or as an additional therapy to treat reactivation or persistent disease after laser therapy (salvage therapy). All parents or guardians were well-informed about the efficacy and possible complications before IVB or IVR, and written informed consent was obtained from each patient's parents or guardians. Anti-VEGF drugs were injected intravitreally with a 30-gauge needle, 0.5–1.0 mm away from the limbus, in the neonatal intensive care unit under topical anesthesia.

### Vitrectomy

Vitrectomy was performed in eyes with vascularly active, progressive stage 4A or worse ROP associated with ROP reactivation. RDs were categorized into three configurations as described by Yonekawa et al. (15): (1) conventional, peripherally elevated ridge- or volcano-shaped stage 5 detachment, (2) midperipheral detachment with tight circumferential vectors, and (3) very posterior detachment with prepapillary contraction. All surgeries were performed by a single surgeon (S.K.). All eyes underwent lens-sparing vitrectomy (LSV) during the initial surgery. The surgical techniques for LSV that were first described by Maguire and Trese (22) in infants were modified as described previously (23). In brief, after conjunctival peritomy, sclerotomies were performed 0.5–1 mm away from the limbus, followed by insertion of 25-gauge or 27-gauge cannulas. The direction of insertion was more posterior than toward the center of the eyeball to avoid lens damage (24). The wide-angle viewing system Resight® (Carl Zeiss Meditec AG, Jena, Germany) was used for the fundus view. Fibrous tissue traction was released to achieve retinal reattachment. Membrane dissection using 25- or 27-gauge horizontal and/or vertical scissors (DORC, Zuidland, Netherlands) was minimized to avoid intraoperative bleeding and/or the creation of an iatrogenic retinal break. For eyes that could not achieve retinal reattachment after the initial vitrectomy, repeated vitrectomies were performed. In patients showing severe fibrous tissue traction who could not be expected to show postoperative retinal reattachment with gas or silicone oil (SO) tamponade, short-term perfluoro-*n*-octane (PFO) tamponade (25) was used. Lensectomy was performed as part of the reoperation, if necessary.

### Statistical Analysis

Statistical analyses were performed using JMP version 14.0, for Windows (SAS Institute, Cary, NC, USA). Data were presented as means and standard deviations, unless otherwise stated. Statistical analyses of continuous variables were performed using Mann-Whitney test. Categorical variables were compared using Fisher's exact test. Statistical significance was set at *P* < 0.05.

### Risk Factors

The potential systemic risk factors obtained from medical records included sex, postmenstrual age (PMA) at birth, birth weight (BW), BW at first fundus examination, Apgar scores (1 and 5 min), history of oxygen inhalation (intubation or nasal inhalation), tracheal intubation, comorbidities (respiratory distress, bronchopulmonary dysplasia, gastrointestinal perforation, patent ductus arteriosus, meconium aspiration syndrome, chorioamnionitis, sepsis, disseminated intravascular coagulation, hydrocephalus, periventricular leukomalacia, and intraventricular hemorrhage), and treatment (erythropoietin administration, red blood cell transfusion, and total parenteral nutrition).

Additionally, the medical records of each eye were reviewed to obtain information regarding the zone of ROP, aggressive ROP (A-ROP) which is defined by the International Classification of Retinopathy of Prematurity, Third Edition as an “rapid development of pathologic neovascularization and severe plus disease without progression being observed through the typical stages of ROP (21), presence of plus disease, previous treatment, PMA at the time of first examination, PMA at the first treatment (laser ablation at the referring hospital or anti-VEGF therapy), PMA at anti-VEGF therapy, and types of anti-VEGF drugs (bevacizumab or ranibizumab).

Patients were divided into two groups: vitrectomy (V) and non-vitrectomy (non-V) groups. The V group included infants who required vitrectomy for RD after anti-VEGF therapy. The non-V group included infants who did not require vitrectomy after anti-VEGF therapy. The demographic and ocular characteristics of the V and non-V groups were compared. In the comparison of demographic characteristics, patients with one eye in the V group and the other in the non-V group were categorized in the V group. For eyes showing reactivation, the period between anti-VEGF therapy and reactivation, PMA at reactivation, and the period between the diagnosis of reactivation and the last examination before reactivation were also reviewed. For eyes in the V group, PMA at the diagnosis of RD, RD configuration, and the vitrectomy procedure were also reviewed.

## Results

A total of 76 eyes of 45 patients with stage 3 ROP who received anti-VEGF therapy were analyzed in this study. All the patients were Japanese. The patient demographics are listed in Table 1. The median follow-up period was 48.3 months (range, 9.4–104.5 months). The median PMA at birth was 24.7 weeks (range, 22.1–29.3 weeks), and the median BW was 591 g (range, 304–1,198 g). The V and non-V groups showed no significant differences in sex, BW, PMA at birth, BW at first examination, Apgar scores (1 and 5 min), or the rates of patients with comorbidities (respiratory distress, bronchopulmonary dysplasia, gastrointestinal perforation, patent ductus arteriosus, meconium aspiration syndrome, chorioamnionitis, sepsis, disseminated intravascular coagulation, hydrocephalus, periventricular leukomalacia, and intraventricular hemorrhage), a history of oxygen inhalation (intubation or nasal inhalation), tracheal intubation, or systemic treatment (erythropoietin administration, red blood cell transfusion, and total parenteral nutrition).

**Table 1:** Demographic characteristics of the study groups.

	**All patients** **(*n =* 45)**	**V group** **(*n =* 5)**	**Non-V group** **(*n =* 40)**	***P***
Boy/Girl	27/18	2/3	25/15	0.339^*^
Birth weight (grams)	625.0 ± 190.0	604.6 ± 118.2	629.0 ± 208.2	0.914^**^
Postmenstrual age (weeks)	24.7 ± 1.5	24.1 ± 1.1	24.8 ± 1.7	0.575^**^
Body weight at first examination (grams)	1,015.1 ± 302.0	976.8 ± 248.5	1021.7 ± 318.8	0.903^**^
Oxygen inhalation (intubation/nasal inhalation)	34 (75.6%)	5 (100%)	29 (72.5%)	0.083^*^
Length of oxygen intake (days)	148.9 ± 117.8	122.8 ± 38.5	153.1 ± 125.9	0.945^**^
Tracheal intubation	13 (28.9%)	2 (40.0%)	11 (27.5%)	0.572^*^
Apgar score at 1 min	3.0 ± 1.9	3.0 ± 1.6	3.0 ± 2.0	0.759^**^
Apgar score at 5 min	5.2 ± 2.1	6.0 ± 2.0	5.1 ± 2.1	0.305^**^
RDS	38 (84.4%)	5 (100%)	33 (82.5%)	0.459^*^
BPD	35 (77.8%)	5 (100%)	30 (75.0%)	0.231^*^
Gastrointestinal perforation	7 (15.6%)	1 (20.0%)	6 (15.0%)	0.899^*^
PDA	28 (62.2%)	4 (80.0%)	24 (60.0%)	0.590^*^
Meconium aspiration syndrome	1 (2.2%)	0 (0%)	1 (2.5%)	0.600^*^
Chorioamnionitis	23 (51.1%)	2 (40.0%)	21 (52.5%)	0.652^*^
Sepsis	8 (17.8%)	2 (40.0%)	6 (15.0%)	0.210^*^
DIC	7 (15.6%)	2 (40.0%)	5 (12.5%)	0.154^*^
Hydrocephalus	4 (8.9%)	1 (20.0%)	3 (7.5%)	0.455^*^
PVL	4 (8.9%)	0 (0%)	4 (10.0%)	0.281^*^
IVH	17 (37.8%)	1 (20.0%)	16 (40.0%)	0.635^*^
Period of EPO administration (days)	46.8 ± 30.5	58.2 ± 38.1	45.1 ± 30.0	0.309^**^
RBC transfusion	34 (75.6%)	4 (80.0%)	30 (75.0%)	0.954^*^
The amount of RBC transfused (mL/kg)	48.2 ± 48.2	40.0 ± 40.0	49.3 ± 50.9	0.871^**^
The period of TPN (days)	32.2 ± 47.8	57.6 ± 94.4	28.2 ± 38.2	0.722^**^

*BPD, bronchopulmonary dysplasia; DIC, disseminated intravascular coagulation; EPO, erythropoietin; IVH, intraventricular hemorrhage; non-V group, the non-vitrectomy group; PDA, patent ductus arteriosus; PVL, periventricular leukomalacia; RBC, red blood cell; RDS, respiratory distress syndrome; TPN, total parenteral nutrition; V group, the vitrectomy group*.

* *Fisher's exact test*.

** *Mann–Whitney U test*.

Thirty and 46 eyes received IVB and IVR, respectively. All eyes showed regression of tortuosity and dilation of the retinal vessels and tunica vasculosa lentis. The ocular characteristics of the patients are presented in Table 2. The two groups showed no significant differences in the ROP zone at the diagnosis of ROP or the ratio of A-ROP. However, the proportion of eyes with plus disease was significantly higher in the V group (50.0%) than in the non-V group (10.0%) (*P* = 0.035). The two groups showed no statistically significant differences in the mean PMA at the first examination, the first treatment, and at anti-VEGF therapy. No systemic or ocular complications related to intravitreal injection were noted, except for reactivation and subsequent RD.

**Table 2:** Ocular characteristics of the study groups.

	**All eyes** **(*n =* 76)**	**V group** **(*n =* 6)**	**Non-V group** **(*n =* 70)**	***P***
A-ROP	10 (13.2%)	2 (33.3%)	8 (11.4%)	0.181^*^
Zone at the diagnosis of ROP				
Zone 1	35 (46.1%)	4 (66.7%)	31 (44.3%)	0.405^*^
Zone 2	41 (53.9%)	2 (33.3%)	39 (55.7%)	
Plus disease	10 (13.2%)	3 (50.0%)	7 (10.0%)	0.035^*^
PMA at the first examination (weeks)	30.5 ± 1.7	30.5 ± 1.7	30.5 ± 1.7	0.922^**^
PMA at the first treatment (weeks)	34.0 ± 1.9	33.3 ± 2.1	34.1 ± 1.9	0.247^**^
PMA at anti-VEGF therapy (weeks)	36.3 ± 3.1	34.3 ± 2.5	36.5 ± 3.1	0.096^**^
VH before anti-VEGF therapy (*n =* 66)	9 (13.6%)	1 (16.7%)	8 (13.3%)	0.821^*^
Treatment				
IVB	30 (39.5%)	3 (50.0%)	27 (38.6%)	0.587^*^
IVR	46 (60.5%)	3 (50.0%)	43 (61.4%)	
Previous Treatment				
Laser	43 (56.6%)	2 (33.3%)	41 (58.6%)	0.232^*^
None	33 (43.4%)	4 (66.7%)	29 (41.4%)	

*A-ROP, aggressive retinopathy of prematurity; IVB, intravitreal injection of bevacizumab; IVR, intravitreal injection of ranibizumab; non-V group, the non-vitrectomy group; PMA, postmenstrual age; VEGF, vascular endothelial growth factor; V group, the vitrectomy group; VH, vitreous hemorrhage*.

* *Fisher's exact test*.

** *Mann–Whitney U test*.

The baseline data of the eyes showing reactivation are presented in Table 3. Reactivation occurred in 27 of 76 eyes (35.1%) 6.4 ± 3.1 weeks after anti-VEGF therapy, including all six eyes in the V group and 21 eyes in the non-V group. The mean period between anti-VEGF therapy and reactivation was 5.7 ± 3.8 and 6.6 ± 3.1 weeks in the V and non-V groups, respectively (*P* = 0.381). The mean PMA at reactivation was 40.0 ± 2.7 and 40.1 ± 4.1 weeks in the V and the non-V groups, respectively (*P* = 0.953). The V group included three of 22 eyes (13.6%) in which the interval between last examination and the diagnosis of reactivation was 1 week or less, and three of five eyes (60.0%) in which the interval was more than 1 week. (*P* = 0.024).

**Table 3:** Ocular characteristics of eyes showing reactivation.

	**All eyes** **(*n =* 27)**	**V group** **(*n =* 6)**	**Non-V group** **(*n =* 21)**	***P***
A-ROP	6 (22.2%)	2 (33.3%)	4 (19.0%)	0.473^*^
Zone at the diagnosis of ROP				
Zone 1	17 (63.0%)	4 (66.7%)	13 (61.9%)	0.830^*^
Zone 2	10 (37.0%)	2 (33.3%)	8 (38.1%)	
Plus disease	7 (25.9%)	3 (50.0%)	4 (19.0%)	0.144^*^
PMA at the first examination (weeks)	30.2 ± 1.7	30.5 ± 1.7	30.2 ± 1.8	0.558^**^
PMA at the first treatment (weeks)	33.3 ± 1.7	33.3 ± 2.1	33.3 ± 1.7	0.682^**^
PMA at anti-VEGF therapy (weeks)	33.7 ± 1.9	34.3 ± 2.5	33.5 ± 1.8	0.726^**^
VH before anti-VEGF therapy (*n =* 25)	4 (16.0%)	1 (16.7%)	3 (15.8%)	0.959^*^
Treatment				
IVB	11 (40.7%)	3 (50.0%)	8 (38.1%)	0.603^*^
IVR	16 (59.3%)	3 (50.0%)	13 (61.9%)	
Previous treatment				
Laser	8 (29.6%)	2 (33.3%)	6 (28.6%)	0.823^*^
None	19 (70.4%)	4 (66.7%)	15 (71.4%)	
Period between anti-VEGF therapy and reactivation (weeks)	6.4 ± 3.1	5.7 ± 3.8	6.6 ± 3.1	0.381^**^
PMA at reactivation (weeks)	40.1± 3.7	40.0 ± 2.7	40.1 ± 4.1	0.953^**^
Examination interval longer than 1 week	5 (18.5%)	3 (50.0%)	2 (9.5%)	0.024^*^

*A-ROP, aggressive retinopathy of prematurity; IVB, intravitreal injection of bevacizumab; IVR, intravitreal injection of ranibizumab; non-V group, the non-vitrectomy group; PMA, postmenstrual age; VEGF, vascular endothelial growth factor; V group, the vitrectomy group; VH, vitreous hemorrhage*.

* *Fisher's exact test*.

** *Mann–Whitney U test*.

**Figure 1:**
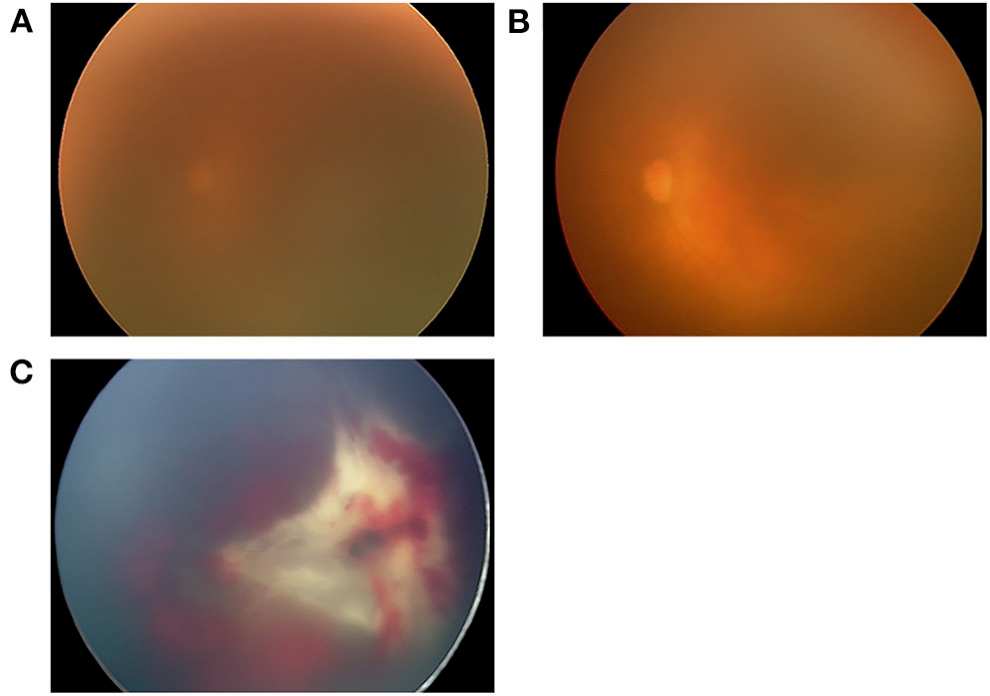
Fundus images of the left eye of one patient in the V group (case 5), who received intravitreal ranibizumab monotherapy (IVR) for zone 1 plus retinopathy of prematurity. After receiving laser therapy for reactivation 7 weeks after IVR, vitreous hemorrhage occurred, and the fundus continued to be invisible for 4 weeks until absorption of the vitreous hemorrhage. **(A)** The fundus image obtained immediately before IVR demonstrated a blurred retina due to a prominent tunica vasculosa lentis. **(B)** Fundus image obtained 2 days after IVR showing improved transparency of the fundus and dilation of the retinal vessels. **(C)** Fundus image obtained 11 weeks after IVR showing volcano-shaped stage 5B ROP with thick proliferative membrane. This eye underwent LSV at 43 weeks postmenstrual age (PMA), and PPV and lensectomy for persistent retinal detachment at 44 weeks PMA, however, it was judged to be inoperable during surgery.

Among the 21 eyes showing reactivation in the non-V group, 17 received laser therapy and four received second anti-VEGF therapy, resulting in regression of the disease in all eyes. The detailed clinical characteristics of the six eyes in the V group are shown in Table 4. In the V group, after reactivation was identified, three eyes received additional laser therapy before the development of RD. RD was first diagnosed 10.1 ± 6.5 (range, 2.6–22.4) weeks after anti-VEGF therapy and subsequent vitrectomy for the treatment of RD was performed 10.9 ± 6.4 (range, 2.9–23.1) weeks after anti-VEGF therapy. The stages of RD in the six eyes in the V group were stage 4A in one eye, 4B in two eyes, 5A in one eye, and 5B in two eyes. The types of RD (15) were conventional (classic) in two eyes and circumferential (unique to RD after anti-VEGF therapy) in four eyes. The mean PMA at vitrectomy was 46.5 ± 7.6 weeks (range, 35.0–56.6 weeks). All six eyes underwent LSV during the first vitrectomy. Three eyes achieved retinal reattachment after the first vitrectomy. Two eyes underwent subsequent lensectomy and vitrectomy with short-term PFO tamponade (25) during the second vitrectomy. One patient showed retinal reattachment after PFO removal. The remaining eye required vitrectomy with SO tamponade for the treatment of RD after PFO removal and showed retinal reattachment after SO removal. One eye underwent PPV and lensectomy as the second surgery; however, it was judged to be inoperable during surgery (Figure 1). Overall, the numbers of vitrectomies were one for 3 eyes, two for 1 eye, three for 1 eye, and four for 1 eye. Finally, all except one eye in the V group showed retinal reattachment at the last examination.

**Table 4:** Clinical characteristics of infants and eyes developing RD after anti-VEGF therapy.

**No**.	**PMA at birth (weeks)**	**Birth Weight, (gram)**	**Eye**	**PMA at initial anti-VEGF (weeks)**	**Zone at the diagnosis of ROP**	**IVB or IVR**	**PMA at reactivation (weeks)**	**Interval between reactivation and the last examination before the reactivation (days)**	**Treatment for reactivation before vitrectomy [PMA (weeks)]**	**PMA at the diagnose of retinal detachment (weeks)**	**Stage and Retinal detachment configuration (26)**	**Procedure of vitrectomy [PMA (weeks)]**	**Final retinal reattachment**
1	24	470	OS	34	1	IVB	38	4	laser [39]	56	S4A: Relatively peripheral with a circumferential configuration	LSV [57]	Yes
2	24	704	OD	35	2	IVB	38	7	None	38	S4B: peripheral ridges	LSV [38]	Yes
3	24	686	OS	37	2	IVR	42	10	None	44	S5B: Relatively peripheral with a circumferential configuration anterior and posterior	LSV [46] PPV+PPL+PFO [52] PFO removal [54]	Yes
4	23	682	OD	32	1	IVR	44	18	laser [39] IVR and laser [43]	44	S5A: Relatively peripheral with a circumferential configuration anterior and posterior	LSV [56] PPV+PPL+PFO [59] PPV+PFO removal+SO [61] SO removal [70]	Yes
5	23	682	OS	32	1	IVR	39	7	laser [39]	43	S5B: Volcano-shaped anterior and posterior	LSV [43] PPV + lensectomy [44]	No
6	22	481	OD	37	1	IVB	39	9	None	43	S4B: Relatively peripheral with a circumferential configuration	LSV [43]	Yes

*IVB, intravitreal injection of bevacizumab; IVR, intravitreal injection of ranibizumab; LSV, lens-sparing vitrectomy; OD, right eye; OS, left eye; PMA, postmenstrual age; PFO, perfluoro-n-octane; PPL, pars plana lensectomy; PPV, pars plana vitrectomy; S4A, stage 4A; S4B, stage 4B; S5A, stage 5A; S5B, stage 5B; SO, silicone oil; VEGF, vascular endothelial growth factor*.

## Discussion

The present study investigated the incidence, features, and treatment outcomes of RD requiring vitrectomy after anti-VEGF therapy in Japanese patients with ROP at a single tertiary referral hospital. The results demonstrated that, of all eyes that received anti-VEGF for ROP, 7.9% eventually developed RD. In addition, plus disease at first examination, as well as a long interval between the last examination prior to reactivation and the diagnosis of reactivation were identified as significant risk factors for the development of RD.

Reactivation of ROP after anti-VEGF therapy in some infants can occur because of a resurgence of VEGF when the anti-VEGF agent is cleared from the eye (27). Such infants may require more than one treatment session (27). To identify high-risk patients and monitor them diligently, several reports have investigated risk factors for reactivation after IVR or IVB. Lower BW, lower gestational age, longer duration of hospitalization, extensive retinal neovascularization, requirement for supplemental oxygen, pre-retinal hemorrhage before injection, younger PMA at treatment, and A-ROP were reported to be possible risk factors for reactivation (7, 9, 12, 13, 20). These individual factors were important for reactivation; however, we found that they were not significant for development of RD after anti-VEGF therapy. On the other hand, plus disease was a possible risk factor for the development of RD after IVR or IVB for the treatment of stage 3 ROP.

Plus disease, which was first defined during the 1980s by an international consensus panel (28) as abnormal posterior pole retinal vessel dilation and tortuosity, is a major indicator for the treatment of severe ROP (21). Eyes with plus disease are likely to show rapid progression of ROP and the development of RD. Biochemical analysis of the vitreous of stage 4 ROP eyes showed significantly elevated VEGF and transforming growth factor-beta (TGF-β) concentrations (29). In addition, studies in adults have demonstrated that the levels of the profibrotic cytokine TGF-β may increase with anti-VEGF therapy (30). TGF-β is a profibrotic cytokine, and upregulation of TGF-β following anti-VEGF therapy might be the cause of tractional RDs in eyes with plus disease receiving anti-VEGF therapy.

Another risk factor for the development of RD after IVR or IVB was the period between the diagnosis of reactivation and the last examination before the reactivation, which was significantly longer in the V group than in the non-V group. This result highlights the importance of close monitoring after anti-VEGF therapy. Although screening criteria for ROP have been established (31), there is no consensus regarding the follow-up of patients treated with anti-VEGF therapy. Martínez-Castellanos et al. (26) recommended that patients who receive IVB should undergo the first follow-up examination at 3–7 days, followed by examinations at 1–2-week intervals based on both the degree of improvement and the stage until complete retinal vascularization. However, frequent visits are often difficult once infants are discharged, especially for infants who may require treatment for other systemic comorbidities. Most previous reports have not described examination schedules or periods between visits after anti-VEGF therapy. On the basis of our findings, whether or not seeing these patients more frequently would have changed the need for vitrectomy. We believe that careful follow-up and early detection of reactivation are critical in reducing the development of RD after anti-VEGF therapy.

With regard to the proportion of cases showing RD after anti-VEGF therapy for ROP, 6 of 76 eyes (7.9%) developed RD in this study. The BEAT-ROP study reported that 2 of 75 eyes (2.7%) developed RD after IVB (4). Another case series found that the incidence of RD was 0–2.0% (10–13). The relatively higher incidence in this study may reflect differences in the timing of anti-VEGF therapy (monotherapy or salvage therapy), types of anti-VEGF drugs (IVB or IVR), variable follow-up schedules, presence or absence of routine additional laser therapy after anti-VEGF therapy, and the degree of immaturity in our patients.

Anti-VEGF crunch syndrome has been described in eyes with proliferative diabetic retinopathy and ROP following anti-VEGF therapy (15, 17, 32). The progression of preexisting tractional RDs after IVB as a surgical adjunct for tractional RDs secondary to proliferative diabetic retinopathy has been reported previously (33). The absence of previous laser photocoagulation and the presence of a ring-shaped fibrovascular membrane were relevant findings in eyes with these IVB-induced complications. RD configurations in this study were classified into three types according to a previous study by Yonekawa et al. (15). In this study, conventional RDs were noted in two eyes (33%), and circumferential RDs were noted in four eyes (67%). None of the eyes developed RDs with pre-papillary configuration, which was noted in 29% of the eyes with or without anti-VEGF therapy in the study by Yonekawa et al. (15). Xu et al. (16) also reported the details of nine eyes that showed RD after anti-VEGF therapy, including three eyes showing conventional RDs and six eyes with circumferential RDs. The proportion of conventional and circumferential RDs and the absence of a prepapillary configuration were similar to our results. RDs with pre-papillary and circumferential configurations have been reported to be difficult to repair, with anatomic success rates of 67 and 75%, respectively (15). In our study, all but one patient with conventional RD achieved retinal reattachment. This variability is likely due to differences in patient populations, small sample sizes, and variable postoperative follow-up periods.

One eye without retinal attachment in our study (Case 5, Figure 1) was diagnosed with stage 5B at 11 weeks after IVR. Before RD was confirmed, the fundus was invisible due to vitreous hemorrhage for 4 weeks. Development of vitreous hemorrhage was likely to be a symptom of increased activity of retinopathy, and earlier vitrectomy was probably desirable considering the risk of RD. The other two cases that required vitrectomy for stages 5B and 5A were cases in which reactivation was found 10 and 18 days after the last examination, respectively. In contrast, in the remaining three eyes, surgical interventions were possible at relatively earlier stages, that is, at stage 4A (one eye) and 4B (two eyes). These patients were followed up with relatively short examination intervals (4, 7, and 9 days). Retinal reattachment was achieved after initial vitrectomy in these eyes. Since the anatomical and functional results of vitrectomy for stage 4 ROP are generally better than those for stage 5 ROP (23), earlier detection of RD and vitrectomy are critical in achieving better surgical results.

This study had several limitations. First, the ROP-related conditions at the time of anti-VEGF therapy, such as the presence or absence of previous treatment before anti-VEGF therapy or follow-up schedules after anti-VEGF therapy, were not uniform, since most of the patients were referred to our hospital and were followed up at the referring hospitals after discharge from our hospital. This could have led to a lack of uniformity in the diagnosis of plus disease and A-ROP. However, our results are likely to reflect real-world clinical data during the period when laser ablation is still the gold standard for primary treatment for ROP. Second, due to the small number of cases with RD, adequate statistical analysis could not be performed. Third, the dosing of IVB (0.25 mg) used in our study was not generalizable, since it was different from the commonly used dosage of 0.625 mg. Lastly, there was a lack of consideration of maternal perinatal comorbidities. Despite these limitations, our study demonstrates that plus disease is a risk factor for the development of RD after anti-VEGF therapy and highlights the importance of close monitoring after anti-VEGF therapy, providing useful information regarding the clinical characteristics of eyes developing RD after anti-VEGF therapy.

In conclusion, nearly 8% of eyes developed RD approximately 10 weeks after anti-VEGF therapy for ROP. The presence of plus disease at the first examination and a long interval between the diagnosis of reactivation and the last examination before reactivation were associated with the development of RD. Careful follow-up with appropriate intervals is recommended after anti-VEGF therapy for ROP.

## Data Availability Statement

The original contributions presented in the study are included in the article/supplementary material, further inquiries can be directed to the corresponding author.

## Ethics Statement

The studies involving human participants were reviewed and approved by the Institutional Review Board of Kindai University Hospital (#26-251). Written informed consent to participate in this study was provided by the participants' legal guardian/next of kin. Written informed consent was obtained from the minor(s)' legal guardian/next of kin for the publication of any potentially identifiable images or data included in this article.

## Author Contributions

CK, SU, YK, and NW acquired the data. CK, SU, CI, and RK analyzed the data and drafted the manuscript. KK and SK revised the manuscript. All authors contributed to conception, design of the research, interpretation of the results, and edited the manuscript.

## Funding

This study was supported by grant-in-aid 20K09800 from the Ministry of Education, Culture, Sport, Science and Technology, Japan. The sponsor or funding organization had no role in the design or conduct of this research.

## Conflict of Interest

The authors declare that the research was conducted in the absence of any commercial or financial relationships that could be construed as a potential conflict of interest.

## Publisher's Note

All claims expressed in this article are solely those of the authors and do not necessarily represent those of their affiliated organizations, or those of the publisher, the editors and the reviewers. Any product that may be evaluated in this article, or claim that may be made by its manufacturer, is not guaranteed or endorsed by the publisher.
